# Sub-massive Pulmonary Embolism and New-Onset Diabetes Mellitus

**DOI:** 10.7759/cureus.64751

**Published:** 2024-07-17

**Authors:** Sapna Rama, Ilya Fonarov, Damian Casadesus

**Affiliations:** 1 Primary Care, Orlando College of Osteopathic Medicine, Orlando, USA; 2 Hospital Medicine, Jackson Memorial Hospital, Miami, USA; 3 Internal Medicine, Jackson Memorial Hospital, Miami, USA

**Keywords:** high-risk pulmonary embolism, arterial hypotension, non-traumatic pulmonary embolism, sub-massive pulmonary embolism, diabetes mellitus

## Abstract

Pulmonary embolism (PE) is a life-threatening disease with variable clinical signs and symptoms, and the diagnosis often requires a high index of suspicion. Patients can have a variety of risk factors that predispose them to venous thromboembolic (VTE) disease. This is a case of a female who presented to the emergency room with new-onset fatigue and shortness of breath for five days. The patient was diagnosed with a sub-massive PE with high-risk features. The patient was also hyperglycemic and diagnosed with new-onset diabetes mellitus. For the PE, she was treated with systemic thrombolysis followed by a standard oral factor Xa inhibitor; for her new onset of diabetes, the patient was started on glargine and lispro insulin. This case underscores the importance of comprehensive management for patients with PE and concurrent metabolic conditions.

## Introduction

Classifying pulmonary embolism (PE) risk is crucial for guiding treatment and improving outcomes. High-risk PE is classified as having sustained systemic arterial hypotension, cardiogenic shock, or the need for pulmonary resuscitation. The in-hospital mortality rate for a high-risk PE is more than 15%. Intermediate-risk PE is classified as having right ventricular dysfunction or positive biomarkers but stable hemodynamics. The in-hospital mortality rate for an intermediate-risk PE is between 3% and 15% [1]. Patients without those findings are classified as low-risk patients with a mortality rate of less than 3%. Untreated PE has an overall mortality rate of up to 30%. Systemic thrombolysis improves hemodynamic parameters and reverses right ventricular (RV) dysfunction; however, it is associated with major bleeding rates of up to 20% [2-4]. 

## Case presentation

A female in her 50s with a history of varicose veins presented to the emergency department with progressively worsening fatigue and dyspnea that started five days before arrival. She denied chest pain, leg swelling, immobilization, recent travel, or surgery. The patient denied using tobacco, alcohol, or recreational drugs. There was no family history of venous thromboembolic (VTE) disease. Upon admission, the vital signs were a blood pressure of 129/91 mmHg, a heart rate of 136 beats per minute, a respiratory rate of 37 breaths per minute, and an oxygen saturation of 95% in ambient air. On physical examination, the lungs were clear to auscultation bilaterally. The cardiovascular exam was significant for tachycardia but at a regular rate. The lower extremities were without edema, erythema, or tenderness. The Homans sign was negative.

Laboratory studies revealed a white blood cell count of 12.0 x 10(3)/L, a hemoglobin count of 14.9 g/dL, a platelet count of 333 x 10(3)/L, serum glucose of 426 mg/dL, and glycosylated hemoglobin of 13.8%. Troponin I was 0.04 ng/mL, and the NT-pro BNP was 813 pg/mL (Table 1).

**Table 1:** The patient's laboratory values NT-pro BNP: N-Terminal pro B-type natriuretic peptide

Investigation	Patient values	Reference values
White blood cell count	12.0 x10(3)/L	4.40 - 10.50 10(3)/L
Platelet count	333x10(3)/L	150-400 × 10(3)/L
Serum glucose	426 mg/dL	70 - 100 mg/dL
Glycosylated hemoglobin	13.80%	4 – 5.6%
Hemoglobin	14.9 g/dL	13.8-17.2 g/dL
Troponin I	0.04 ng/mL	<0.04 ng/mL
NT-pro BNP	813 pg/mL	<100 pg/mL

**Figure 1:**
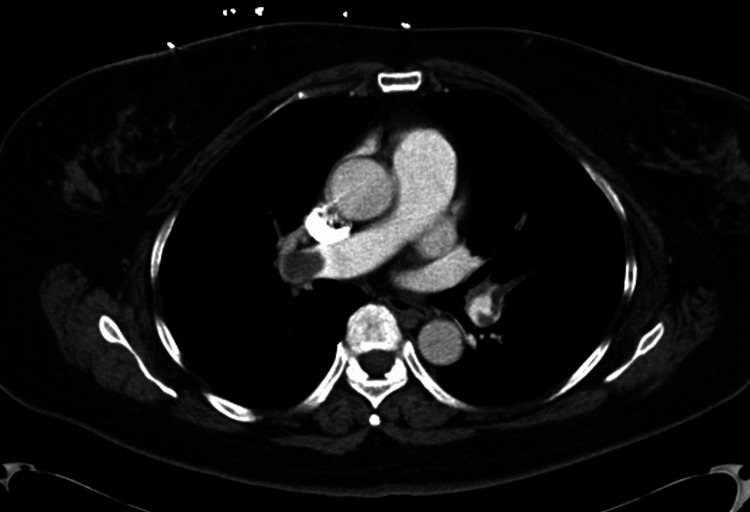
A computed tomography angiogram of the chest (axial images) shows pulmonary embolisms at the distal right pulmonary artery extending into the right interlobar pulmonary artery and segmental branches.

**Figure 2:**
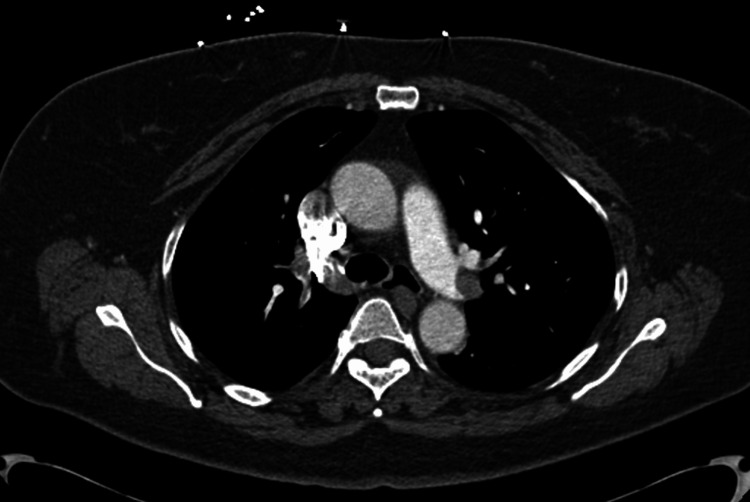
A computed tomography angiogram of the chest (axial images) shows a left-sided pulmonary embolism.

**Figure 3:**
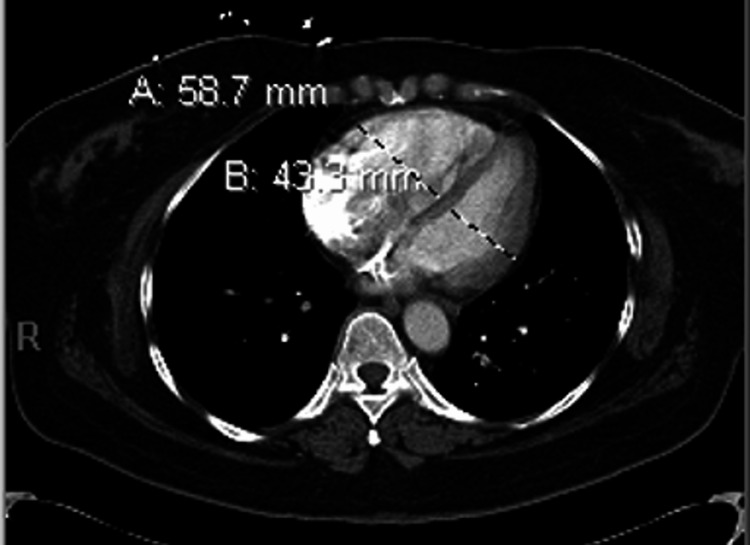
A computed tomography angiogram of the chest (axial images) shows an asymmetric enlargement of the right ventricle compared to the left and mild flattening of the interventricular septum, suggesting right heart strain.

In the emergency room, a point-of-care echocardiogram was performed as well as a CT angiography (CTA) of the chest with contrast. The CTA scan of the chest with contrast revealed extensive, large bilateral PEs extending from the bilateral distal right and left main pulmonary arteries to segmental branches (Figures 1, 2). Imaging also showed that the heart was mildly enlarged, with asymmetric enlargement of the right ventricle compared to the left ventricle and mild flattening of the interventricular septum, suggesting right heart strain (Figure 3).

**Figure 4:**
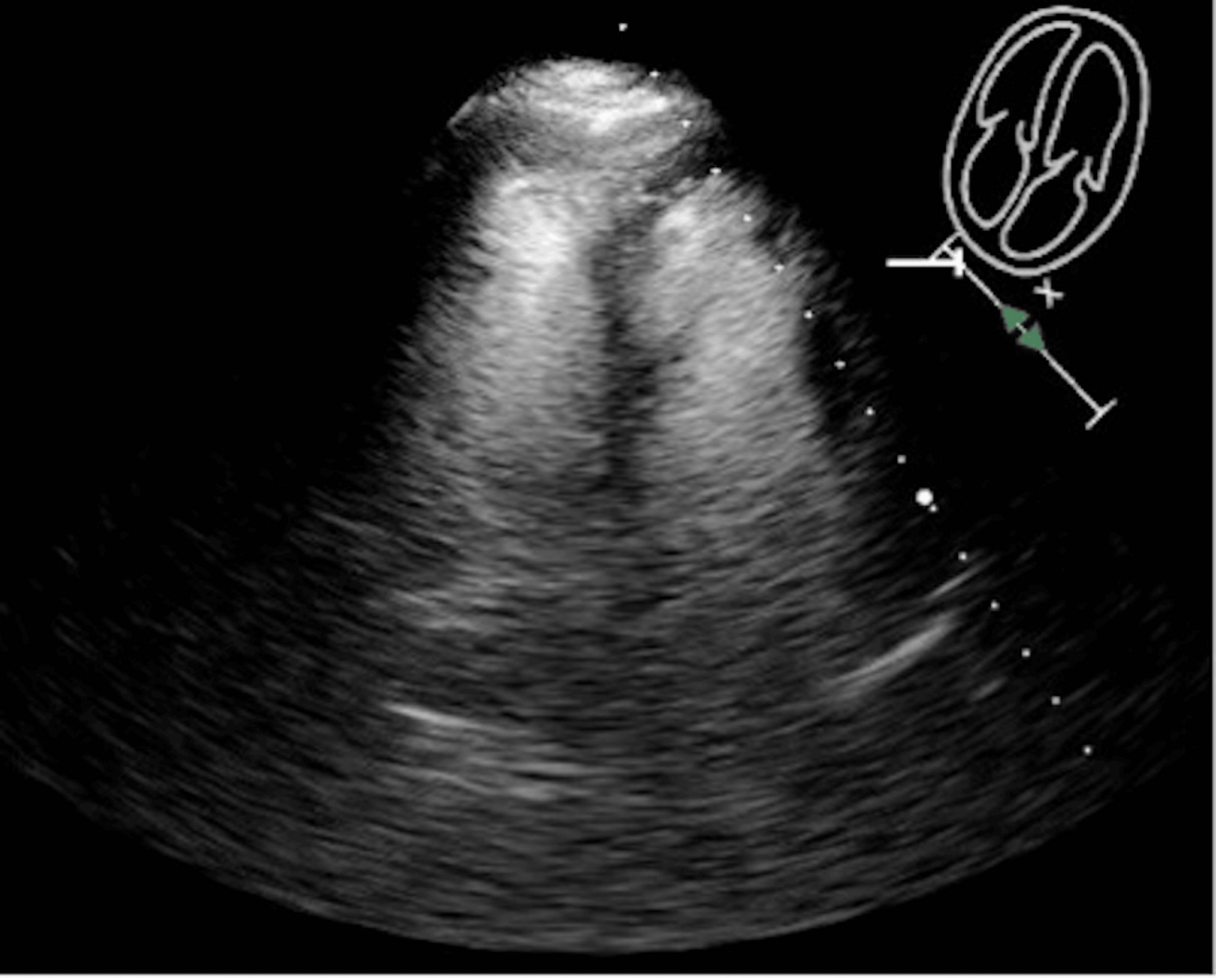
On echocardiography with contrast (four chamber view), hypokinesis of the lateral right ventricular wall and preserved apical contractility consistent with the McConnell sign is noted.

The echocardiogram revealed right heart strain, and there was hypokinesis of the lateral right ventricular wall and preserved apical contractility consistent with the McConnell sign, which may be seen in acute PE (Figure 4). Doppler ultrasound did not show evidence of venous thrombosis in the deep veins of the lower extremities.

The patient was diagnosed with a sub-massive PE with high-risk features. The patient was administered alteplase 10 mg bolus, followed by a 40 mg infusion over two hours. This was recommended by the institution's Pulmonary Embolism Response Team. The patient was started on glargine insulin 22 units at bedtime and lispro insulin six units with meals, as well as a lispro insulin sliding scale. She was placed on supplemental oxygen of 2 liters by nasal cannula and admitted to the intensive care unit.

The patient had a great response to the treatment, with improvements in fatigue and dyspnea over the first 24 hours, so supplemental oxygen was discontinued. Upon discharge, the patient’s symptoms had completely resolved, blood glucose had improved, and the patient had no complications. The patient was discharged on bedtime glargine insulin, metformin 1000 mg by mouth twice daily, and glipizide 5 mg by mouth daily. The patient was discharged 72 hours after admission.

The patient was followed by her primary care physician a few months later. The patient's glycemic control improved with a glycohemoglobin A1C of 5.9% from 13.8%. Consequently, metformin and glargine insulin were discontinued, and glipizide 5 mg was titrated down to once daily. The patient had no other thrombotic recurrences.

## Discussion

Dyspnea and chest pain associated with tachycardia are the most common presenting symptoms of PE. Normotensive patients with acute PE and RV dysfunction are classified as having sub-massive PE and have an increased risk of adverse events. They make up about 20%-30% of PE cases.

Sub-massive PE treatment with systemic thrombolysis is controversial. Patients experiencing clinical deterioration with sub-massive PE may be considered for thrombolysis [5]. It has shown not only rapid resolution of major pulmonary emboli but also a decrease in pulmonary artery pressure and improvement of RV function and left ventricular output [6]. A meta-analysis of high-risk PE patients revealed a significant decrease in recurrence or death, dropping from 19% in patients using heparin monotherapy to 9.4% in patients treated with thrombolysis [7]. A meta-analysis by Jaff et al. found that there was a statistically non-significant rise in major bleeding complications with systemic thrombolysis compared to heparin alone (10.1 vs. 7.3%) [8]. The use of systemic thrombolytic treatment has shown a reduction in all-cause mortality and hemodynamic collapse within seven days over standard anticoagulation; however, it comes at the cost of an increased risk of non-intracranial major bleeding and intracranial bleeding. In over two-thirds of high-risk PE cases, systemic thrombolysis was not administered. The percentage of unstable patients with pulmonary embolism receiving thrombolysis decreased from 40% to 23% from 1999 to 2008 in the USA [9].

Our patient had an uncommon presentation of weakness and hyperglycemia. However, she was diagnosed in a timely manner with a sub-massive PE with high-risk features and new-onset diabetes mellitus. Diabetes mellitus is associated with hypercoagulability via activation of the coagulation system [10]. In addition, they have impaired fibrinolysis, elevated levels of coagulation activation markers, and many clotting factors [11]. According to a meta-analysis by Bai et al., diabetics are at increased risk of VTE [12]. The association between VTE risk and DM in metanalysis is not always consistent. Patients with DM may have other comorbidities and confounding factors that place them at higher risk for VTE [13]. Diabetes is associated with a greater risk of adverse events and in-hospital mortality in patients with a PE [14]. In our patient, systemic thrombolysis was used without complications, with clinical improvement, and without recurrence of VTE after a three-month follow-up.

## Conclusions

This case report underscores the importance of the association between diabetes and a higher VTE risk. Patients with new-onset diabetes and cardiorespiratory symptoms should be considered for further evaluation for VTE. Prompt recognition and early intervention would favor a better outcome. Future research should investigate the biological mechanisms, conduct large-scale epidemiological studies, identify predictive biomarkers, and foster interdisciplinary collaboration to understand the association between venous thromboembolic disease and diabetes. 
